# Artificial Intelligence and Algorithmic Bias in Burn Care: A Literature Review of Ethical Challenges

**DOI:** 10.1093/jbcr/irag105

**Published:** 2026-06-24

**Authors:** Joshua Khorsandi, Abu-Bakr Ahmed, Jason Mirharooni, Michael Kahen, Joshua Ahdout, Demitri Franzoni, Joshua MacDavid

**Affiliations:** Department of Plastic and Reconstructive Surgery, Kirk Kerkorian School of Medicine at University of Nevada Las Vegas, Las Vegas, NV 89106, United States; Department of Plastic and Reconstructive Surgery, Kirk Kerkorian School of Medicine at University of Nevada Las Vegas, Las Vegas, NV 89106, United States; Department of Medicine, Florida International University Herbert Wertheim College of Medicine, Miami, FL 33199, United States; Department of Plastic and Reconstructive Surgery, Kirk Kerkorian School of Medicine at University of Nevada Las Vegas, Las Vegas, NV 89106, United States; Department of Medicine, Touro University Nevada, Las Vegas, NV 89014, United States; Department of Plastic and Reconstructive Surgery, University of Nevada Las Vegas, Las Vegas, NV 89106, United States; Department of Plastic and Reconstructive Surgery, University of Nevada Las Vegas, Las Vegas, NV 89106, United States

**Keywords:** artificial intelligence, burn care, algorithmic bias, health equity, fairness auditing

## Abstract

Artificial intelligence (AI) is increasingly integrated into burn care for triage, burn-depth assessment, prognostic scoring, pain management, and telemedicine-enabled resource allocation. These tools promise greater efficiency and precision, yet raise substantial ethical concerns regarding transparency, accountability, and bias. This narrative review synthesizes literature from 2010 to 2025 on AI, predictive modeling, and digital tools in burn care, supplemented by evidence from critical care, emergency medicine, radiology, dermatology, and oncology. Systematic searches of PubMed, Embase, and Scopus identified studies that reported algorithm development or deployment and discussed or allowed inference about equity, fairness, or interpretability. Across medical domains, algorithms frequently misclassified outcomes for racial and ethnic minorities, socioeconomically disadvantaged patients, women, older adults, and individuals with complex comorbidities, while burn-specific models rarely evaluated subgroup performance or reported demographic composition. Common problems included unrepresentative datasets, opaque modeling pipelines, and the absence of formal bias audits. Ethical analyses were fragmented and seldom grounded in established frameworks of biomedical ethics or AI governance. This review argues that current trajectories risk embedding and amplifying inequities in an already vulnerable burn population. It proposes concrete strategies for fairness-oriented design, reporting, validation, and post-deployment monitoring, emphasizing justice, nonmaleficence, transparency, accountability, and stakeholder engagement. Responsible adoption of AI in burn care will require moving beyond technical performance alone toward explicit attention to equity and ethical safeguards throughout the model life cycle.

## INTRODUCTION

Burn injuries remain a major global health burden, with disproportionate impact in low- and middle-income countries and among marginalized populations. Accurate burn depth and total body surface area assessment, early prognostication, and triage to specialized centers are vital but difficult in resource-constrained settings, motivating interest in artificial intelligence (AI) to augment clinician judgment and standardize burn care.^1^

Artificial intelligence and machine learning support diagnosis, risk prediction, imaging interpretation, and workflow optimization.^2^ In burn care, early work emphasized computer-assisted burn size estimation and telemedicine consultation via smartphone image capture sent to burn specialists with decision-support recommendations.^3^ More recently, convolutional neural networks, multispectral imaging, and imaging–electronic health record platforms automate burn-depth assessment and wound-healing prediction, often with high accuracy in controlled datasets.^4^ Parallel work refines mortality prediction and prognostic scores using burn registries (eg, the Bochum Burn Survival [BoBS] score).^5^

Artificial intelligence-enabled tools are also being explored for pain management, rehabilitation, and follow-up, including virtual reality (VR)-based analgesia and telemedicine/remote wound monitoring, with growing interest in scalable outpatient applications across diverse settings.^3^ Other fields show that AI may reproduce or exacerbate inequities. Bias has been documented across racial, ethnic, and socioeconomic lines, and models can divert resources when trained on biased data or optimized for proxy targets such as cost; a commercial population health algorithm underestimated risk among Black patients by using healthcare spending as the label.^6^^,^^7^ Bias also appears in pulse oximetry, dermatology imaging, and emergency department/intensive care unit (ICU) prediction tools, motivating fairness frameworks.^8^

Importantly, algorithmic bias in healthcare AI does not arise solely from flawed software design, but often reflects underlying structural inequities embedded within clinical data and healthcare systems. Machine learning models trained on datasets that underrepresent racial and ethnic minorities, patients with darker skin tones, uninsured individuals, or populations from low-resource settings may systematically perform less accurately for these groups. In addition, the use of proxy variables such as healthcare utilization, cost, or prior access to care can unintentionally encode disparities into predictive systems, leading to unequal recommendations for triage, treatment intensity, or resource allocation. Because burn care frequently relies on imaging, prognostic scoring, and time-sensitive decision-making, these biases may disproportionately affect already vulnerable populations and contribute to widening disparities in outcomes.

Burn care heightens the stakes of algorithmic bias: patients face pain, trauma, long recoveries, and disability risk, and severe burns are overrepresented among children, unhoused people, migrants, and rural or low-resource populations. Errors in prognostication or triage can delay transfer, drive inappropriate surgical decisions, or misallocate scarce intensive care resources, yet burn AI models often prioritize global performance over equity or fairness.^1^

Despite rapid technical advances, the burn care literature has not systematically examined AI through the lens of algorithmic bias, equity, and ethical governance. Most burn-related AI studies emphasize predictive performance while providing limited reporting on dataset representativeness, subgroup performance, or downstream clinical implications, and ethical considerations are often fragmented or implicit. This literature review addresses this gap by synthesizing burn AI, predictive modeling, and digital tools through an explicit ethical and equity-focused lens. Drawing on burn-specific studies and comparative evidence from other clinical domains, it examines sources of bias across the AI life cycle and proposes practical strategies for fairness-oriented design, validation, deployment, and post-deployment monitoring to support responsible adoption of AI in burn care.

## METHODS

A structured narrative literature review was conducted using PubMed, Embase, and Scopus (accessed December 30, 2025). Search strategies combined controlled vocabulary and free-text terms related to burn injury (“burn,” “thermal injury,” “scald,” “flame injury”), AI and predictive modeling (“machine learning,” “deep learning,” “neural network,” “predictive model,” “risk score,” “decision support”), and digital health modalities (“telemedicine,” “smartphone,” “virtual reality,” “digital wound assessment”). To capture broader ethical and fairness considerations, supplementary searches included terms such as “algorithmic bias,” “healthcare disparities,” “fairness,” “equity,” “race,” “ethnicity,” “sex,” “gender,” “socioeconomic,” “explainability,” and “interpretability.” The search was limited to English-language, peer-reviewed literature published from 2010 to 2025. For contextual depth, earlier high-impact studies addressing foundational issues in medical AI ethics or algorithmic bias were retained when they remained influential to contemporary practice.

Eligible burn-related studies included original research or systematic reviews that developed, validated, or evaluated AI or machine-learning models, predictive tools, digital wound assessment systems, telemedicine decision-support platforms, or VR applications for acute care or rehabilitation. Articles from adjacent clinical domains were included when they contained explicit analyses of demographic performance differences, fairness metrics, or broader ethical implications relevant to clinical decision-making. Exclusion criteria eliminated non-English publications, conference abstracts without full text, purely technical computer science papers, and general digital health studies lacking substantive discussion of ethics or bias.

Title and abstract screening was followed by full-text review to confirm eligibility. Because the review emphasized ethical and fairness considerations rather than quantitative synthesis, no minimum sample size was required, although very small pilot studies were interpreted cautiously. Data extraction captured clinical context, data sources, model characteristics, target outcomes, and performance metrics.

Two reviewers independently coded the selected literature using a structured abstraction framework that was applied consistently to capture ethical, fairness, and bias-related concepts. Discrepancies in code assignment or thematic interpretation were resolved through iterative discussion until consensus was achieved, with additional re-review of the source article when needed. The deductive component applied predefined ethical domains to each study, whereas the inductive component allowed new concepts and recurring concerns emerging from the literature to be incorporated into the coding structure. Themes were subsequently synthesized across studies through comparative analysis to identify recurring ethical patterns, differences between burn-specific and non-burn applications, and underrepresented areas requiring further investigation.

Ethical and fairness issues were identified through a combined deductive–inductive coding process using predefined constructs—such as dataset representativeness, demographic reporting, subgroup performance assessment, use of sensitive attributes, label quality, transparency, interpretability, governance, and accountability—supplemented by emergent themes including contestability, clinician override, and post-deployment monitoring. Thematic synthesis compared patterns within burn-specific studies and across external medical domains to identify convergences, gaps, and persistent challenges in addressing algorithmic bias and ethics in clinical AI. The final review comprised 20 studies that met all inclusion criteria and were synthesized to examine ethical, fairness, and bias-related considerations in AI-enabled burn care and related clinical domains.

## RESULTS

### Overview of the burn AI and digital health literature

The search identified a growing corpus of work on AI and digital tools in burn care, particularly in burn-depth assessment, wound imaging, prognostic scoring, telemedicine, and VR-based pain management.^1^ Most studies emphasized model development and internal validation, often reporting global accuracy, sensitivity, specificity, or area under the receiver operating characteristic curve. Relatively few articles provided detailed descriptions of dataset demographic composition, and even fewer presented stratified performance metrics or fairness analyses by race, ethnicity, sex, age, socioeconomic status, or comorbidity burden. When ethical issues were mentioned, they tended to be confined to brief comments on the potential to improve access or the importance of clinician oversight, without systematic engagement with equity, bias, or governance. Table 1 provides a concise evidence map of burn-care AI and digital health applications, summarizing major use cases, typical data sources and settings, and the most common gaps in demographic reporting and subgroup performance evaluation.

**Table 1:** Burn-AI Evidence Map and Equity-Reporting Gaps

Application area	Typical data + settings	What’s missing (equity/reporting gap)	References
Burn-depth assessment and wound monitoring (CNN’s, multispectral/thermography, EHR-linked tools)	Photo/MSI/thermography ± clinical variables; often single-center cohorts	Rare reporting of skin tone/Race/ethnicity; almost no stratified performance for darker skin	4, 9, 10
Prognostic scoring and complication prediction (mortality, sepsis/AKI, LOS; registry ML; BoBS)	Burn registries EHR; higher resource burn centers	Limited demographic composition; stratified performance by race/SES/ age rarely shown; minimal triage/resource-allocation equity analysis	5, 11
Telemedicine + VR (teleburn apps, remote wound monitoring; VR analgesia/rehab)	Smartphone images + connectivity; VR trials usually small, tertiary centers	Digital divide not evaluated (device /bandwidth /literacy); limited demographic diversity and subgroup	3, 12, 13

Abbreviations: AI, artificial intelligence; AKI, acute kidney injury; BoBS, Bochum Burn Survival score; CNN, convolutional neural network; EHR, electronic health record; LOS, length of stay; ML, machine learning; MSI, multispectral imaging; SES, socioeconomic status; VR, virtual reality.

### AI for burn-depth assessment and wound monitoring

A substantial subset of burn-focused AI studies addressed burn-depth classification or wound-healing prediction using imaging modalities such as conventional color photography, multispectral imaging, structured light, thermography, or ultrasound. Convolutional neural networks and other deep learning architectures achieved high reported accuracy in distinguishing superficial from deep burns or predicting the need for grafting.^4^ Some systems integrated imaging data with clinical variables in electronic health records to generate real-time decision support within the bedside workflow.^9^

Despite these advances, almost no burn imaging studies reported performance separately for patients with darker skin tones or for distinct racial and ethnic groups. Many training datasets were drawn from single-center cohorts in predominantly White or lighter-skinned populations in Europe or North America, or from regional registries with limited demographic diversity.^1^ This omission is concerning given evidence from dermatology and other photo-based diagnostics that both human and AI systems perform less accurately on darker skin and that training sets are often heavily skewed toward lighter skin types.^10^

Similarly, smartphone-based wound assessment applications and teledermatology-like systems for burns rarely documented differential image quality or usability by patient age, socioeconomic status, or geographic location, even though access to high-end smartphones, lighting, and bandwidth is unevenly distributed and may correlate with social disadvantage.^3^ Few studies examined whether automated segmentation or severity scoring was equally reliable in images captured by lay caregivers compared with trained clinicians, raising the possibility that tools marketed as bridging access gaps could inadvertently deliver less accurate assessments to those in greatest need.

### Prognostic scoring, complication prediction, and resource allocation

Several studies applied machine learning to improve prognostic models in burn care, including mortality risk prediction, prediction of graft surgery, length of stay, or complications such as sepsis, inhalation injury, and acute kidney injury.^11^ Work using the German Burn Registry and other large datasets evaluated random forests, gradient boosting, and ensemble methods, sometimes developing new scores such as the BoBS score that sought to retain clinical interpretability while harnessing machine learning to refine variable weighting.^5^ Studies generally reported improved discrimination compared with older scores such as the Baux or revised Baux scores, particularly in contemporary cohorts where supportive care has evolved.^11^

Yet, even in these relatively data-rich prognostic studies, demographic reporting and fairness analyses were limited. Many papers reported mean age and sex distribution but did not describe racial or ethnic composition, socioeconomic indicators, or comorbidity profiles in sufficient detail to assess representativeness. Stratified performance metrics by race, sex, or age were rarely presented. There was little discussion of how model performance might differ between high-resource and low-resource settings, despite the frequent use of registry data from well-resourced burn centers. Only a few studies explicitly considered the implications of integrating prognostic outputs into triage or resource-allocation decisions, and none systematically examined whether machine learning–based scores might differentially prioritize some patients over others.^11^

### Pain management, rehabilitation, and patient experience

Virtual reality–based systems for procedural pain relief during dressing changes or rehabilitation sessions represent another prominent area of digital innovation in burn care. Systematic reviews and randomized trials indicate that immersive VR can reduce self-reported pain and anxiety and improve the procedural experience for adult and pediatric patients with burn injuries.^12^ While not always framed as AI, many of these systems incorporate adaptive features, gamification, or real-time monitoring that may rely on algorithmic personalization.

The equity dimensions of such interventions were rarely explored. Studies often recruited small samples from single tertiary centers, with limited demographic diversity and minimal reporting of race, ethnicity, socioeconomic status, or language.^13^ Few trials examined differential acceptability or effectiveness by age, sex, cultural background, or digital literacy. Moreover, the cost and infrastructural requirements for VR hardware and software were seldom considered in relation to burn centers serving under-resourced populations. As VR-based interventions move from research to routine practice, these omissions could translate into disparities in access to nonpharmacologic pain management and rehabilitation tools.

### Telemedicine, triage, and remote care

Telemedicine and remote monitoring tools have been promoted as strategies to expand access to burn expertise, especially in rural or resource-limited settings. Smartphone-based consultation apps, teleburn programs linking community hospitals with burn centers, and remote wound monitoring platforms have shown feasibility for initial assessment, follow-up, and rehabilitation.^3^ In parallel, emergency medicine and trauma literature describes AI-supported triage systems that use clinical and textual data to prioritize emergency department patients, with growing interest in language models as general-purpose triage engines.^14^

Across these domains, the fairness implications of algorithmic triage and tele-consultation have been unevenly addressed. Emergency department studies increasingly acknowledge that conventional triage is vulnerable to racial, ethnic, and language-based biases and that AI systems could either mitigate or exacerbate these inequities depending on design and deployment.^15^ However, burn telemedicine articles rarely examine whether access to, and use of, remote consultation tools differs by geography, socioeconomic status, or race, or whether image quality and connectivity issues systematically disadvantage certain groups.^3^ There is virtually no burn-specific literature evaluating how algorithmic triage or remote decision-support tools perform across demographic subgroups or how they might influence decisions about transfer to a burn center versus local management.

### Comparative evidence from other specialties on algorithmic bias

In contrast to the relatively sparse burn-specific fairness analyses, a robust body of literature in other specialties documents algorithmic bias and inequities. Systematic reviews and policy analyses show that healthcare algorithms can perpetuate or exacerbate racial and ethnic disparities in access, quality, and outcomes, depending on how they are trained and deployed.^6^ Obermeyer et al.’s study of a commercial population health algorithm revealed that using healthcare costs as a proxy outcome led to systemic underestimation of risk among Black patients, who received fewer resources despite comparable or greater illness burden.^7^

Critical care and emergency medicine research highlights technology-driven inequities in diagnostic and monitoring tools. Bias in pulse oximetry accuracy across skin pigmentation, for example, has prompted regulatory reconsideration and underscores how seemingly simple devices can systematically misestimate physiologic status in people with darker skin.^8^ Studies of sepsis prediction algorithms and ICU testing practices show that disparities in laboratory ordering, such as lactate measurements, can propagate bias into AI models and contribute to higher mortality among non-White patients.^16^

In radiology and dermatology, deep learning systems sometimes exhibit reduced performance for darker skin tones and underrepresented groups, even when global accuracy appears high.^10^ These findings have catalyzed calls for demographic performance reporting, explicit fairness metrics, and the use of explainable AI methods to understand error patterns. Similarly, reviews and frameworks in digital health ethics emphasize the need for fairness, accountability, transparency, and stakeholder engagement throughout the AI model life cycle.^17^

Across these external domains, a central theme is that algorithmic bias often emerges from a combination of unrepresentative data, problematic proxy outcomes, opaque feature selection, and deployment in structurally inequitable settings. These mechanisms are highly relevant to burn care, yet the burn AI literature has only begun to engage with them in a systematic way.

## DISCUSSION

**Figure 1:**
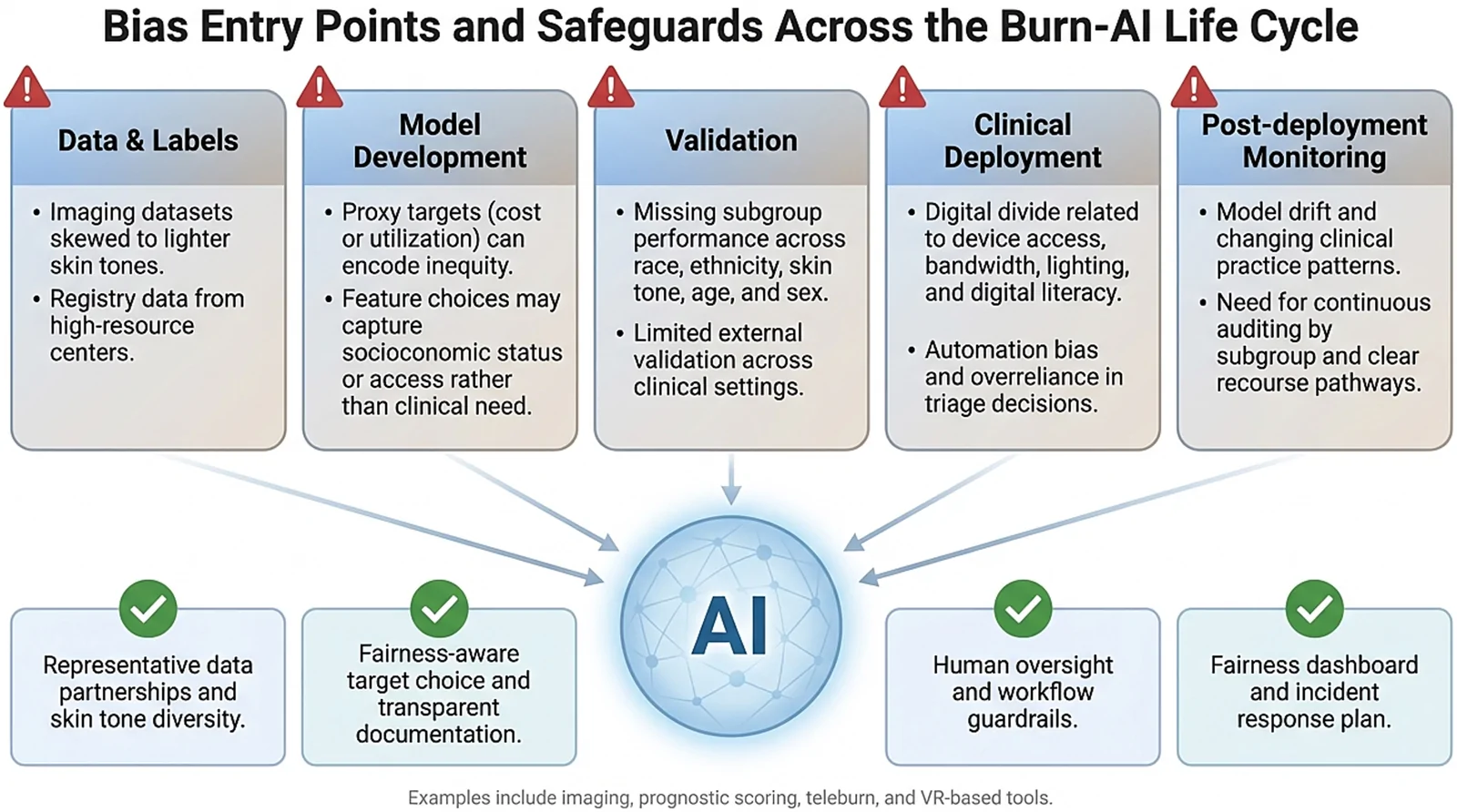
Bias Entry Points and Safeguards Across the Burn-AI Life Cycle. Conceptual overview of how inequities can arise from data/labels, model development, validation, deployment, and post-deployment monitoring, with mitigation strategies relevant to burn AI tools. Image created by authors using FigureLabs. Abbreviation: AI = artificial intelligence.

This review shows that AI and digital tools in burn care have advanced significantly in technical sophistication but lag in explicit evaluation and mitigation of algorithmic bias. Figure 1 illustrates key points in the burn-AI life cycle at which algorithmic bias may arise, alongside corresponding safeguards to promote fairness, transparency, and accountability. Although deep learning models for burn-depth assessment, machine learning–based prognostic scores, telemedicine platforms, and VR interventions demonstrate promising performance and feasibility, equity considerations are seldom systematically incorporated into their development or reporting.

### Mechanisms of inequity: dataset composition and label quality

A primary pathway through which AI may generate inequities in burn care is the composition of training and validation datasets. Burn registries and single-center datasets often overrepresent patients treated at high-resource tertiary centers in high-income countries, who may differ substantially from patients in rural, low-resource, or conflict-affected settings where burn incidence is high.^1^ If models are trained predominantly on populations that are younger, insured, or from majority racial groups, they may systematically misestimate risk or misclassify burn depth for older adults, uninsured patients, or racial and ethnic minorities. Similar concerns apply to imaging datasets in which most photographs depict lighter skin tones, raising the possibility that models will be less accurate for patients with darker skin, as already documented in dermatology AI.^10^

Label quality further complicates fairness. Ground truth for burn depth is often derived from clinical observation, delayed healing outcomes, or histology in selected cases. Interobserver variability in burn-depth assessment is well recognized, and the accuracy of bedside classification may itself differ by patient characteristics such as skin tone, age, and comorbidity.^1^ If clinicians are systematically more likely to underestimate depth in certain groups, these biases can become embedded as labels in the training data, leading AI models to replicate and reinforce inequitable patterns. Analogously, predictive models trained on healthcare utilization or costs rather than disease burden can encode structural disparities in access to care, as demonstrated in population health algorithms.^7^

### Feature selection, target choice, and model architecture

Feature engineering and target selection also influence fairness. Prognostic models in burn care often incorporate variables such as age, total body surface area burned, and inhalation injury, which are clinically meaningful.^11^ However, models may also include proxies for socioeconomic status, access to care, or comorbidity coding practices, such as insurance type, length of prehospital delay, or prior hospitalization frequency. If such features are correlated with structural disadvantage, optimizing for predictive performance without considering equity can reproduce inequitable outcomes, for example, by deprioritizing patients whose prior access to care has been limited.

Burn AI tools that optimize resource utilization, such as algorithms designed to identify high-cost or “high-risk” patients for intensive case management, warrant particular scrutiny. Lessons from the Obermeyer study suggest that using cost-based targets can allocate additional resources preferentially to groups that already receive more care, exacerbating disparities.^7^ Burn centers that deploy models predicting readmission risk or length of stay should carefully consider whether the chosen targets and features reflect need, opportunity to benefit, and equity, rather than efficiency alone.

Model architecture interacts with fairness through interpretability and transparency. Complex deep learning models may capture subtle nonlinear relationships at the expense of interpretability, making it difficult to detect biased patterns without dedicated fairness audits and explainable AI techniques.^17^ Although simpler models such as logistic regression or interpretable scoring systems like BoBS can be more transparent, they are not inherently fair; their fairness still depends on data, features, and targets. The choice between more complex or more interpretable models should therefore be guided not only by overall accuracy but also by the capacity to understand and monitor subgroup performance.

### Deployment context, digital divides, and structural conditions

Even a relatively fair model at the time of development can produce inequitable outcomes when deployed in structurally unequal environments. Teleburn systems that rely on smartphones, high-bandwidth connectivity, and stable electricity may preferentially benefit patients with greater digital access, leaving behind those in rural or impoverished communities.^3^ Remote wound-monitoring apps that require patients to capture and upload high-quality images may place additional burdens on individuals with limited health literacy, cognitive impairment, or mobility limitations, potentially widening gaps in follow-up care.

Similarly, integrating AI-based burn-depth assessment into high-resource centers equipped with multispectral imaging or thermography may improve local care while doing little for centers without such technology. Resource allocation decisions that rely on prognostic algorithms may amplify disparities if they are used to rationalize denial of intensive therapies to patients deemed to have a low likelihood of benefit based on biased models. These concerns illustrate that fairness must be assessed not only at the level of algorithmic performance but also in relation to broader systems of access, reimbursement, and clinical workflow.

### Ethical principles and AI governance in burn care

The ethical challenges outlined above intersect with core principles of biomedical ethics and emerging AI governance frameworks. Justice requires that benefits and burdens of new technologies be distributed fairly and that existing inequities not be exacerbated. Nonmaleficence obliges clinicians and developers to avoid foreseeable harms, including systematically misclassifying or undertreating vulnerable groups. Beneficence supports the use of AI when it genuinely improves outcomes and experiences, such as relieving pain through VR or expediting accurate burn assessment.^18^

Transparency and explainability are central to both autonomy and accountability. Patients and clinicians should understand, at least at a high level, how AI-assisted recommendations are generated and what their limitations are. Standards such as TRIPOD + AI emphasize complete and clear reporting of model development and validation, which is a prerequisite for evaluating risk of bias and applicability.^19^ Accountability demands that clear lines of responsibility exist for monitoring model performance, addressing harms, and revising or withdrawing tools when they are found to be inequitable. Frameworks such as GUIDE and national guiding principles for addressing algorithmic bias in healthcare stress the need for explicit fairness criteria, documentation of trade-offs, and mechanisms for recourse.^20^

Stakeholder engagement, including input from burn survivors, caregivers, and communities disproportionately affected by burns, is essential to ensure that AI systems align with patient values and priorities.^2^ For example, communities may value explainability and human oversight over marginal gains in predictive accuracy, or they may prioritize equitable access to VR-based pain control even if robust data on long-term outcomes are still emerging.

### Strategies to mitigate bias in burn-related AI tools

Translating these ethical principles into practice requires concrete strategies throughout the AI life cycle in burn care. Table 2 summarizes fairness-oriented safeguards across the burn-AI life cycle, linking common bias risks in data, model development/validation, and deployment to practical mitigation actions and governance checks.

**Table 2:** Fairness-Oriented Safeguards Across the Burn-AI Life Cycle

Life-cycle stage	Bias risk (what goes wrong)	Safeguard to require (what to do + what to check)	References
Data + labels	Unrepresentative cohort; biased “Ground truth” (clinical depth levels vary by skin tone/setting)	Collect/merge diverse datasets; report demographics/skin tone; label audits + interrater reliability; document missingness by subgroup	1, 10
Development + validation	Strong global metrics hide subgroup failure; opaque models limit detection	Prespecify subgroup analyses (race/ethnicity/sex/age/SES/skin tone); report calibration + error rates by subgroup; external validation in different sites/resources	11, 17, 19
Deployment + monitoring + governance	Drift and workflow inequities; telehealth tools disadvantage low-access groups; unclear accountability	Post-deployment fairness dashboard; monitor adverse outcomes/transfer delays by subgroup; clinician override + resource; oversight review of intended use	6, 17, 20

Abbreviations: AI, artificial intelligence; SES, socioeconomic status.

First, representative data collection must be prioritized. Developers should actively seek datasets that include diverse racial and ethnic groups, a wide range of skin tones, children and older adults, and patients from varied socioeconomic and geographic backgrounds. Where local demographics are skewed, collaborations across centers and countries, as well as targeted oversampling of underrepresented groups, may be necessary to achieve sufficient diversity.^1^

Second, fairness and performance reporting standards should be integrated into burn AI research. Authors should routinely report dataset demographic composition and evaluate model performance across key subgroups such as race, ethnicity, sex, age, comorbidity burden, and, for imaging algorithms, skin tone. Reporting guidelines like TRIPOD + AI, together with equity-focused frameworks such as GUIDE, can be adapted to include burn-relevant fairness metrics and subgroup analyses.^19^

Third, external validation and transportability assessment must extend beyond traditional performance metrics to include fairness. Burn prognostic models and imaging algorithms should be tested in sites that differ from the development setting in patient mix, resource levels, and clinical practices. Particular attention should be paid to model behavior in low-resource environments, in populations with higher proportions of darker skin tones, and in centers serving socioeconomically marginalized groups.^11^

Fourth, continuous post-deployment auditing is essential. Once AI tools are integrated into clinical workflows, ongoing monitoring should track performance and error rates across demographic groups, including whether delays in transfer, misclassification of burn depth, or adverse outcomes cluster among specific populations. Quality improvement infrastructure within burn centers can incorporate fairness dashboards and feedback loops to clinicians and developers.^17^

Fifth, multidisciplinary ethics and governance structures should oversee AI adoption in burn care. Ethics committees, institutional review boards, and specialized AI oversight bodies can review proposed uses of prognostic algorithms, telemedicine triage tools, and VR interventions, considering equity implications alongside safety and efficacy. These bodies can also help establish policies on clinician oversight, such as when and how clinicians may override algorithmic outputs, and on mechanisms for patients and families to contest decisions influenced by AI.^6^

**Figure 2:**
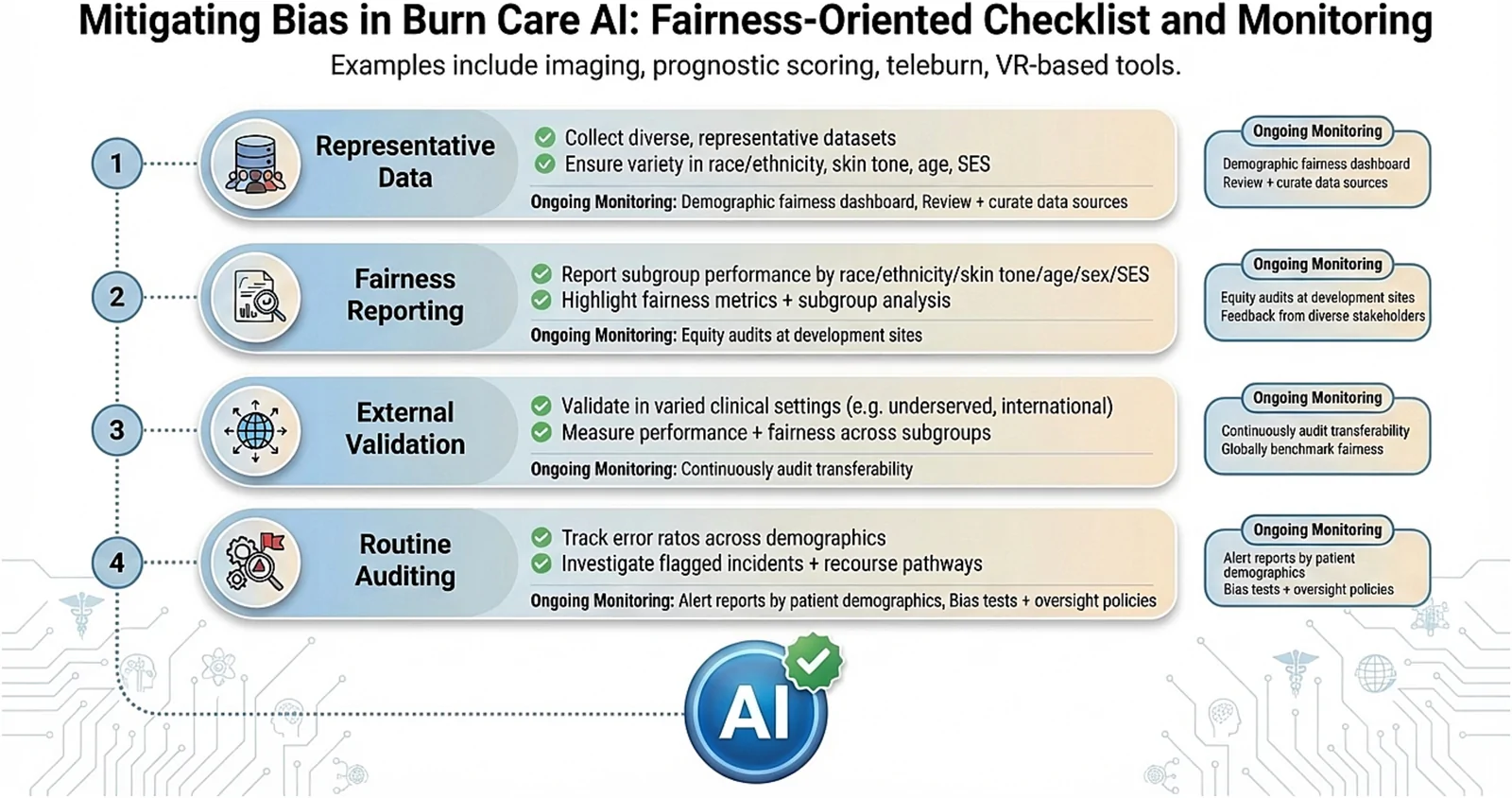
Mitigating Bias in Burn Care AI: Fairness-Oriented Checklist and Monitoring. Practical steps for representative data collection, subgroup reporting, external validation, and routine auditing with ongoing demographic monitoring. Image created by authors using FigureLabs. Abbreviations: AI = artificial intelligence; SES = socioeconomic status; VR = virtual reality.

Finally, clinician education and cultural change are necessary complements to technical fixes. Burn care teams need training to interpret algorithmic outputs critically, recognize potential biases, and engage in shared decision-making with patients that incorporates but does not defer entirely to AI recommendations.^2^ Without such training, there is a risk of overreliance on AI or, conversely, blanket distrust that prevents beneficial tools from being used. Figure 2 summarizes a practical, fairness-oriented checklist and monitoring framework to support responsible implementation of burn care AI, reinforcing the need for continuous oversight alongside clinician education and judgment.

## CONCLUSION

Artificial intelligence could transform burn care by improving depth assessment, prognostic scoring, telemedicine, and pain management. Although early models show strong technical promise, experience from other specialties warns that AI can reinforce inequities if justice, transparency, and accountability are overlooked. Burn care has conducted little systematic fairness testing despite the vulnerability of its patients. Progress requires representative data, subgroup reporting, external validation, ongoing auditing, and strong governance. With thoughtful design and oversight, AI could help reduce, rather than worsen, disparities in burn care.

## Notes

Author contributions: Joshua Khorsandi (Writing—original draft [lead]), Abu-Bakr Ahmed (Data curation, Formal analysis [lead]), Jason Mirharooni (Writing—review & editing [lead]), Michael Kahen (Methodology [equal]), Joshua Ahdout (Visualization [equal]), Demitri Franzoni (Supervision, Writing—review & editing [equal]), and Joshua MacDavid (Supervision, Writing—review & editing [equal])

Funding: This research received no external funding.
